# Thalamocortical Functional Connectivity in Patients With White Matter Hyperintensities

**DOI:** 10.3389/fnagi.2021.632237

**Published:** 2021-03-18

**Authors:** Chen Chen, Xiaojing Wang, Shanshan Cao, Jun Zhang, Zhiqi Wang, Wen Pan, Jinying Yang, Yanghua Tian, Bensheng Qiu, Qiang Wei, Kai Wang

**Affiliations:** ^1^Department of Neurology, The First Affiliated Hospital of Anhui Medical University, Hefei, China; ^2^Anhui Province Key Laboratory of Cognition and Neuropsychiatric Disorders, Hefei, China; ^3^Department of Neurology, The Second Affiliated Hospital of Anhui Medical University, Hefei, China; ^4^Laboratory Center for Information Science and Technology of China, Hefei, China; ^5^Collaborative Innovation Center of Neuropsychiatric Disorders and Mental Health, Hefei, China; ^6^Hefei National Lab for Physical Sciences at the Microscale and the Centers for Biomedical Engineering, University of Science and Technology of China, Hefei, China; ^7^The College of Mental Health and Psychological Sciences, Anhui Medical University, Hefei, China; ^8^Institute of Artificial Intelligence, Hefei Comprehensive National Science Center, Hefei, China

**Keywords:** cognition, thalamocortical, functional connectivity, resting-state, white matter hyperintensities

## Abstract

**Background:** White matter hyperintensities (WMH)s is a very common neuroradiological manifestation in the elderly and is an increased risk of dementia and cognitive decline. As we all know, the thalamocortical circuit plays an important part in cognition regulation. However, the role of this circuit in WMHs and its related cognitive deficits is still unclear.

**Method:** Eighty WMH patients and 37 healthy controls (HCs) were enrolled in the current study. WMH patients were divided into a mild WMH group (*n* = 33) and moderate-severe WMH group (*n* = 47) according to Fazekas scores. Resting-state functional magnetic resonance imaging (rs-fMRI) data of all participants were collected for thalamocortical functional connectivity (FC) analysis. The analysis was performed in two steps. First, the whole cerebral cortex was divided into six regions of interest (ROIs), which were used as seeds to investigate the changes of FC with the thalamus. Then, the subregion of the thalamus generated in the previous step was used as the seed for FC analysis with the whole brain.

**Results:** In the first step of FC analysis, it was found that precentral gyrus (PrCG)-interthalamic adhesion (ITA) FC values in moderate-severe WMH group were higher than those in HC and mild WMH groups. However, when compared with the HC group, the increase of PrCG-ITA FC values in mild WMH group was not statistically significant. In the second step of FC analysis, the ITA was set as the seed, and compared with the HC group, the results showed that the FC values of the ITA-medial frontal gyrus (MFG) in mild group and moderate-severe WMH groups were significantly increased. In addition, the FC values in moderate-severe group were significantly higher than those in mild group. Finally, it was also found that FC values (PrCG-ITA and ITA-MFG) were significantly correlated with neuropsychological test results for multiple cognitive functions such as memory, execution and attention in WMH patients.

**Conclusion:** Abnormal thalamocortical FC was closely related with cognitive impairments in WMH patients.

## Introduction

White matter hyperintensities (WMH)s is the main imaging features of cerebral small vessel disease. Specifically, they are defined as hyperintensities on a T2-weighted image in the periventricular or subcortical deep white matter, and are common in older individuals with cognitive decline (D'Arbeloff et al., 2019). Furthermore, WMHs increase risk of stroke, dementia and death (Debette and Markus, 2010; Xu et al., 2018). However, the pathogenesis of cognitive impairments of WMH remains uncertain.

Recent studies have suggested that the thalamus is involved in the regulation of cognitive function. Thalamic nuclei can be divided into two categories according to its inputs: first order and higher order (Guillery, 1995). In first order nuclei relay systems, several subregions of the thalamus receive input from surrounding sensory organs and subcortical structures and, in turn, send projections to primary sensory and motor cortex regions (Bickford et al., 2015). At the same time, in the higher-order system, several other subregions of the thalamus receive most of the inputs from the cortex directly, thereby regulating the transmission of information between the broader cortical regions related to the cognitive process (Saalmann et al., 2012; Ward, 2013). Given that the driving input to these nuclei originates from the cortex itself, and the projections of the thalamocortical are more dispersed than the first-order relay network, while higher-order nuclei also play an important role in regulating cortical activity and coordinating activities between cortical regions (Sherman, 2016). Previous studies have shown that there are significant connectivities between the thalamus and brain regions which closely related to different functions, such as the frontal, temporal, parietal and occipital lobes (Behrens et al., 2003; Johansen-Berg et al., 2005; Zhang et al., 2008, 2010). There is a study showed that patients with a higher burden of WMH showed lower default mode network functional connectivity in the thalamus. Moreover, the increase in the average dispersion of the white matter tract between thalamus and the posterior cingulate cortex is an independent risk factor for slower processing speed in patients with schizophrenia (Chen P. et al., 2019). In summary, these evidence suggests that the thalamocortical circuit may be involved in the cognitive impairments associated with WMH.

Functional magnetic resonance imaging (fMRI) is a prominent tool that helps in non-invasive examinations. Using resting-state fMRI (rs-fMRI), researchers found that the brain emitted consistent low frequency fluctuations in the range 0.01–0.08 Hz, and that these frequencies could be used to indicated intrinsic activity within the whole brain (Biswal, 2012). rs-fMRI relies on spontaneous low frequency fluctuations in the blood oxygen level dependent signal. Functional connectivity (FC) is the study of the interaction between two different brain regions. FC analysis based on regions of interest (ROI) is the most common method for investigating functionality in the brain. In general, many studies have used resting-state FC analysis to identify thalamocortical circuits in various disorders (Acharya et al., 2019; Chen X. et al., 2019; Ye et al., 2019).

In this study, we hypothesized that the thalamocortical FC may play an important role in the mechanism of cognitive impairments in WMH. We used rs-fMRI to measure thalamocortical FC in two steps: first, the cortex was segmented into six ROIs (Brown et al., 2017; Wei et al., 2020), which were regarded as a seed to locate FC changes in the thalamus. Next, the identified thalamic subregion in the first step were set as the seed to locate FC changes in the whole cortex.

## Methods

### Subjects

Eighty WMH patients were recruited in the Department of Neurology, the First Affiliated Hospital of Anhui Medical University (China). Based on the Fazekas scale (Kester et al., 2014), patients with WMHs were further divided into a mild (*n* = 33, Fazekas score 1–2) and moderate-severe group (*n* = 47, Fazekas score 3–6) (Morotti et al., 2020; Zhu et al., 2020). Severity of WMH was quantified using the periventricular and deep WMH Fazekas scores from 0 to 3 (Grade 0, no WMH; Grade 1, focal or punctate lesions; Grade 2, beginning of confluent lesions; and Grade 3, confluent lesions) (Fazekas et al., 1987). The UBO detector is used to extract and calculate variables in the WMH area (available for download at https://cheba.unsw.edu.au/group/neuroimaging-pipeline). It is a reliable, efficient and completely automatic segmentation process to generate WMH volumes and output the number of WMH cluster in periventricular areas, deep and lobar WMH regions, arterial areas, and even in whole brain (Jiang et al., 2018). UBO Detector is also used as a convenient tool in this study to quantitatively describe WMH volume of mild and moderate-severe group.

The criteria for WMH patient enrollment were: (1) aged 50–80 years; (2) magnetic resonance suggesting the presence of white matter damage; and (3) no contraindications to MRI. Exclusion criteria included: (1) internal and external cranial artery stenosis > 50% or previous carotid surgery; (2) subcortical or cortical infarct lesions with a diameter of more than 1.5 cm; (3) craniocerebral space-occupying lesions, intracranial hemorrhage, craniocerebral trauma or history of surgery; (4) WMHs caused by metabolic and hereditary leukoencephalopathy or immune-mediated inflammatory demyelinating diseases including multiple sclerosis, optic neuromyelitis or acute diffuse encephalomyelitis; and (5) hearing and visual impairments that impeded neuropsychological testing. According to Fazekas classification (source of interpretation), the WMH group was divided into a mild WMH and moderate-severe WMH group, with 39 and 54 individuals, respectively.

Healthy control (HCs) had no history of neurological or psychiatric diseases, or drug and alcohol abuse. HCs were able to cooperate with neuropsychology background tests and magnetic resonance examination. Data requirements for rs-fMRI: excessive motion in the scanner was <2 mm in any direction and <2.0° in any angular motion.

All participant signed informed consent, and the study was approved by the Anhui Medical University Ethics Committee.

### Neuropsychological Examination

A neuropsychological examination was performed on all participants the same day as the MRI examination, including mental status and global cognitive assessments.

#### Overall Cognition Assessment

AD-8 questionnaire was used to assess change in functional performance secondary to cognitive change (Hendry et al., 2019). The Montreal Cognitive Assessment (MoCA) was also used to assess the overall cognitive assessment of WMH patients. There is a study that had confirmed the validity, reliability and clinical applicability of the MOCA scale in patients with cerebral small vessel disease (Wong et al., 2009).

#### Single Cognition and Psychiatric Symptoms Assessment

Anxiety disorders were assessed using the Generalized Anxiety Disorder 7 Scale (Hinz et al., 2017). Use Patient Health Questionnaire 9 (Smarr and Keefer, 2011) to evaluate depression symptoms (Smarr and Keefer, 2011). The Chinese auditory vocabulary learning test (CAVLT) was used to evaluate the participants' verbal memory skills. The Stroop color word test (SCWT) assesses the ability of participants to inhibit cognitive interference (Stroop, 1935). The trail making test (TMT), consisting of two parts (TMT-A and TMT-B), was used as an indication of organic brain damage (Reitan, 1958), and cognitive flexibility is the TMT primary executive function. The verbal fluency test (VFT) requires participant naming as many examples of animals as possible in 60 s, it activates multiple cognitive processes (Troyer et al., 1997). Digital span (DS) was used to evaluate the ability to focus the mind, anti-interference and transient memory, reflecting the participant's attention function (Ostrosky-Solís and Lozano, 2006). The symbol digital modalities test (SDMT) is a digital replacement test which was used to determine distraction, perception speed, visual scanning speed and tracking function (Smith, 1973; Lewandowski, 1984). The Boston naming test (BNT), which one of the most widely used tests for assessing naming ability and lexical retrieval was also utilized (LaBarge et al., 1986).

### MRI Data Acquisition

The MRI scan was completed at the University of Science and Technology of China (Hefei, China), and MRI images were acquired on a General Electric HD 750 w 3.0 T MRI scanner with an 8-channel head-coil (General Electric, Waukesha, WI, USA). During the scan, all subjects were told to keep relax, keep their eyes closed and stay awake. The subjects were equipped with tight but comfortable foam pads to minimize head movement, and they were equipped with earplugs to reduce the noise of the scanner. The T1-weighted imaging sequence is: slice thickness = 5 mm and total surface area = 20 layers. T2-weighted imaging sequences: layer thickness = 5 mm and total layer = 20 layers. The rs-fMRI scans were conducted with the following parameters: repetition time = 2,400 ms; echo time = 30 ms; flip angle = 90°; matrix size = 64 × 64, field of view = 192 × 192 mm^2^; slice thickness = 3 mm; and 46 continuous slices (one voxel = 3 × 3 × 3 mm^3^). Acquire T1-weighted images through a brain volume (BRAVO) sequence with 188 slices (TR = 8.16 ms; TE = 3.18 ms; flip angle = 12°; field of view = 256 × 256 mm^2^; slice thickness = 1 mm; and voxel size = 1 × 1 × 1 mm^3^).

### Functional Data Preprocessing

Functional magnetic resonance data were analyzed using Data Processing and Analysis for Brain Imaging (Yan et al., 2016) and statistical parametric mapping software package was used for preprocessing (Izquierdo-Garcia et al., 2014). Initially, the first ten volumes of the scanning process are discarded to achieve stable longitudinal magnetization and adapt the participants to the noisy scanning environment, and then underwent modifications in the following order: slice timing correction; realignment; normalization by diffeomorphic anatomical registration through exponentiated lie algebra; and transfer of the functional images to the Montreal Neurological Institute space (3 × 3 × 3 mm^3^) based on the transformation matrix and white matter and cerebrospinal fluid signals. Twenty-four head motion parameters were removed and used as a useless covariate. The image was smoothed with the full width (the half-maximum value is 4 mm), and the higher motion time point (defined as a frame-wise displacement > 0.5), the prior time point, and the latter two time points were scrubbed (Power et al., 2012). Finally, the time points were detrended and band-pass filtered (0.01–0.08 Hz).

### Cortical ROI-to-Thalamus FC Analysis

**Figure 1:**
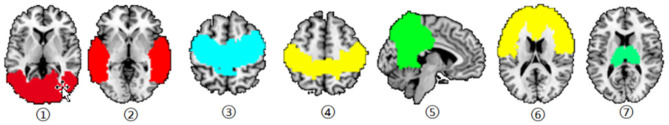
Six cortical ROIs and the thalamus used in functional connectivity analysis. Occipital gyrus; Temporal gyrus; Precentral gyrus; Postcentral gyrus; Parietal gyrus; Frontal gyrus; and Thalamus.

First, the cerebral cortex was divided into six brain regions, including the frontal lobe, precentral gyrus (PrCG), postcentral gyrus, temporal lobe, occipital lobe and parietal lobe (Xu et al., 2018) (Figure 1). The thalamus is spatially defined by the Harvard-Oxford atlas (http://fsl.fmrib.ox.ac.uk/fsl/fslwiki/Atlases) (Figure 1) and these ROIs were used as seeds in the analysis to identify the functional connection between the cortical ROIs and each voxel in the thalamus. Then, calculate the Pearson correlation coefficient between the average blood oxygenation level dependent (BOLD) signal of each cortical ROI and the BOLD signal of each voxel in the thalamus. Apply Fisher's z-transform to the correlation value to ensure the normality. After that, the FC values of the brain regions generated in FC analysis were extracted and the correlation with the scores of the neuropsychological examination score were investigated. Then, calculate the Pearson correlation coefficient between the average BOLD signal of each cortical ROI and the BOLD signal of each voxel in the thalamus. Apply Fisher's z-transform to the correlation value to ensure normality.

### Thalamus-to-Whole Brain FC Analysis

The clusters in the thalamus generated from thalamocortical FC analysis were then used as new ROI. FC analysis of thalamus-to-whole brain was performed among the three groups. In short, for each subject and each thalamic ROI, a correlation map is created by calculating the Pearson correlation coefficient between the average BOLD signal of the thalamic ROI and the BOLD signal of all voxels in the brain. Then, use Fisher's r-z transformation to convert these correlation graphs into Z-value graphs. Finally, the FC values were extracted and the correlation with the scores of the neuropsychological examination scores were investigated.

### Statistical Analysis

Use SPSS 19.0 (SPSS, Inc., Chicago, IL, USA) to analyze the demographic information and neuropsychological background scores among three groups. All data were tested for normality. Normal distribution data is expressed as mean ± standard deviation, and non-normal distribution data is expressed as median and interquartile range. The differences among the three groups in regard to demographic, clinical, and neuropsychological data were compared using one-way analysis of variance, chi-square (χ^2^) tests, or the Kruska-Wallis test in cases of non-normality. Finally, Pearson's correlation analysis was used to analyze the relationship between the FC value and neuropsychological test scores of each of the participants. *p* < 0.05 was considered statistically significant. Multiple comparisons of these analyses were corrected using the Gaussian random field (significant at levels of voxel *p* < 0.001 and cluster *p* < 0.05).

## Results

### Demographic and Neuropsychological Results

There was no statistically significant difference in sex, age and education level among the three groups. Except for hypertension, there was no significant difference among the three groups in diabetes, hyperlipidemia and smoking and alcohol intake (*p* > 0.05). There were more hypertension patients in the mild WMH group than in the HC group (χ^2^ = 11.887, *p* = 0.001), while hypertension in the moderate-severe WMH group was significantly higher than in the HC group (χ^2^ = 25.076, *p* < 0.001). The moderate-severe WMH group performed significantly worse in MOCA, CAVLT, SDMT, TMT, and BNT tests compared with the HC group. The CAVLT scores of the moderate-severe WMH group were significantly lower than those in the mild WMH group (*p* < 0.05). In the TMT and BNT experiments, the mild WMH group had significantly lower scores than the HC group (*p* < 0.05). Demographic information and neurological background test results of the three groups are shown in Table 1.

**Table 1:** Demographic data and neuropsychological background test results of the three groups.

	**Moderate-Severe^**a**^ WMH (*n* = 47)**	**Mild WMH^**b**^ (*n* = 33)**	**HC^**c**^ (*n* = 37)**	**F/χ^2^**	***p***
Age (years)	63.06 ± 7.29	62.87 ± 8.61	60.24 ± 6.99	1.655	0.196
Male	27	19	17	0.67	0.514
Education (years)	8.06 ± 3.63	8.43 ± 4.29	8.81 ± 3.57	0.399	0.672
Hypertension	32	18	11	6.27	***0.003***
Diabetes	6	5	3	0.869	0.648
Hyperlipidemia	10	10	9	0.854	0.653
Smoking	16	15	9	3.462	0.177
Drinking	18	8	10	2.154	0.341
PHQ-9	3 (1.9)	3.5 (1, 5.75)	2 (0.5)	0.996	0.373
GAD-7	2 (0.5)	2 (0,4)	1 (0,4)	0.634	0.533
AD-8	2 (1.4)	2 (1, 3.75)	3 (2,4)	1.458	0.237
MOCA	19.84 ± 4.73	21.54 ± 4.16	22.24 ± 3.79	3.433	***0.036***
VFT	14.40 ± 4.66	15.30 ± 5.16	16.65 ± 4.02	2.169	0.12
CAVLT-Learning memory	6.77 ± 1.97	7.75 ± 2.29	7.78 ± 2.22	2.731	***0.07***
CAVLT-interference	3.47 ± 2.06	2.92 ± 1.85	2.96 ± 2.29	0.781	0.461
CAVLT-Immediate recall	5.63 ± 3.48	8.70 ± 4.00	9.03 ± 2.54	11.94	***<0.001***
CAVLT-delayed recall	6.14 ± 3.11	8.07 ± 3.07	8.80 ± 2.65	6.738	***0.002***
CAVLT-recognition	11.57 ± 3.54	13.03 ± 2.26	13.67 ± 1.53	5.762	***0.004***
SDMT-duration number	19.39 ± 8.96	22.85 ± 11.91	24.62 ± 9.83	2.42	***0.094***
SDMT-correct number	25.42 ± 10.59	29.92 ± 12.55	33.25 ± 8.30	5.136	***0.008***
DS-forward	6.43 ± 1.50	6.50 ± 1.43	6.66 ± 1.49	0.243	0.785
DS-backward	3.65 ± 1.14	4.03 ± 1.54	3.93 ± 0.96	0.966	0.384
SCWT-dot	23.97 ± 11.13	22.21 ± 8.96	20.50 ± 5.25	1.309	0.275
SCWT-word	30.54 ± 17.04	28.96 ± 12.35	24.57 ± 5.47	1.891	0.157
SCWT-interference	43.06 ± 18.86	39.49 ± 15.89	37.73 ± 10.43	1.053	0.353
SCWT	12.51 ± 10.38	10.53 ± 10.49	13.17 ± 7.96	0.605	0.548
TMT-A	86.89 ± 32.49	81.59 ± 39.65	59.90 ± 18.90	7.155	***0.001***
TMT-B	174.65 ± 76.98	155.27 ± 77.06	133.15 ± 42.00	3.386	***0.038***
BNT	13.02 ± 1.53	12.92 ± 1.43	13.87 ± 1.23	4.329	***0.016***

a *Represents the Moderate-Severe WMH group*.

b *Represents the Mild WMH group*.

c *Represents the HC group*.

*Hypertension: P (a-c) < 0.001, P (b-c)=0.016; MOCA: P (a-c) = 0.013; CAVLT-learning: P (a-c) = 0.045; CAVLT-immediate recall: P (a-c) < 0.001, P (a-b) < 0.001; CAVLT-delay recall: P (a-c) = 0.001, P (a-b) = 0.016; CAVLT-recognition: P (a-c) = 0.002, P (a-b) = 0.032; SDMT-duration number: P (a-c) = 0.034; SDMT-correct number: P (a-c) = 0.002; TMT-A: P (a-c) < 0.001; P (b-c) = 0.008; TMT-B:P (a-c) = 0.011; BNT: P (a-c) = 0.012, P (b-c) = 0.012; PHQ-9, patient health questionnaire-9; GAD-7, generalized anxiety disorder-7; AD-8, Alzheimer's dementia questionnaire-8; MOCA, Montreal cognitive assessment; VFT, verbal fluency test; CAVLT, Chinese auditory verbal learning test; SDMT, symbol digit modalities test; DS, digit span; SCWT, Stroop color and word test; TMT, trail making test; BNT, Boston naming test; WMH, white matter hyperintensities; p-values < 0.05 are labeled in bold and italic*.

### Cortical ROI-to-Thalamus FC Results

**Figure 2:**
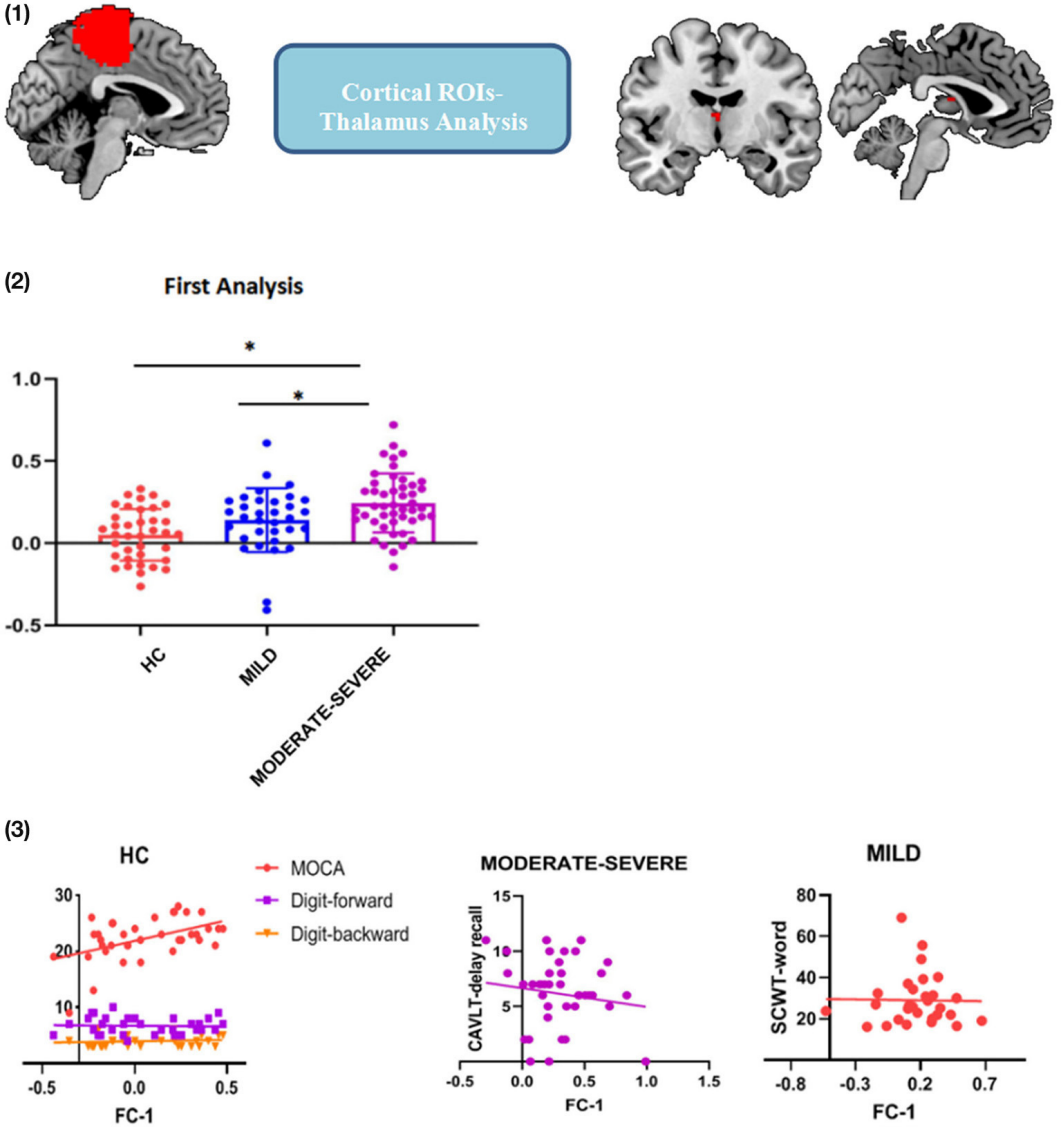
Functional connectivity between the precentral gyrus ROI and ITA. (1) PrCG-ITA detected in cortical ROIs-to-thalamus analysis; (2) FC strength of PrCG-ITA among the three groups; (3) Correlation between cognitive assessment scores and PrCG-ITA FC strength. FC, functional connectivity; PrCG, precentral gyrus; ITA, interthalamic adhesion; MOCA, Montreal cognitive assessment; CAVLT, Chinese auditory verbal learning test; SCWT, Stroop color and word test; WMHs, white matter hyperintensities; HC, healthy controls. *p < 0.05; error bars represent standard deviation.

The first FC analysis indicated that PrCG- interthalamic adhesion (ITA) FC values were different among the three groups [Figure 2: (1)]. We found that the PrCG-ITA FC values of the moderate-severe WMH group were significantly higher than those of the mild WMH and HC groups (*t* = 2.399, *p* = 0.019 and *t* = 4.042, *p* < 0.001, respectively); however, there was no statistical difference between the mild WMH and HC groups [*t* = 1.592, *p* = 0.115; Figure 2: (2)]. Pearson's correlation analysis showed that in the HC group, the PrCG-ITA FC values were positively correlated with MOCA, DS-forward and DS-backward scores (MOCA: *r* = 0.485, *p* = 0.002; DS-forward: *r* = 0.413, *p* = 0.016; DS-backward: *r* = 0.346, *p* = 0.049). In mild WMH group, the PrCG-ITA FC values were positively correlated with the STROOP-word score (*r* = 0.394, *p* = 0.035), whereas in the moderate-severe WMH group, PrCG-ITA FC values were positively correlated with CAVLT-delay recall [*r* = 0.338, *p* = 0.029; Figure 2: (3)].

### Thalamus-to-Whole Brain FC Results

**Figure 3:**
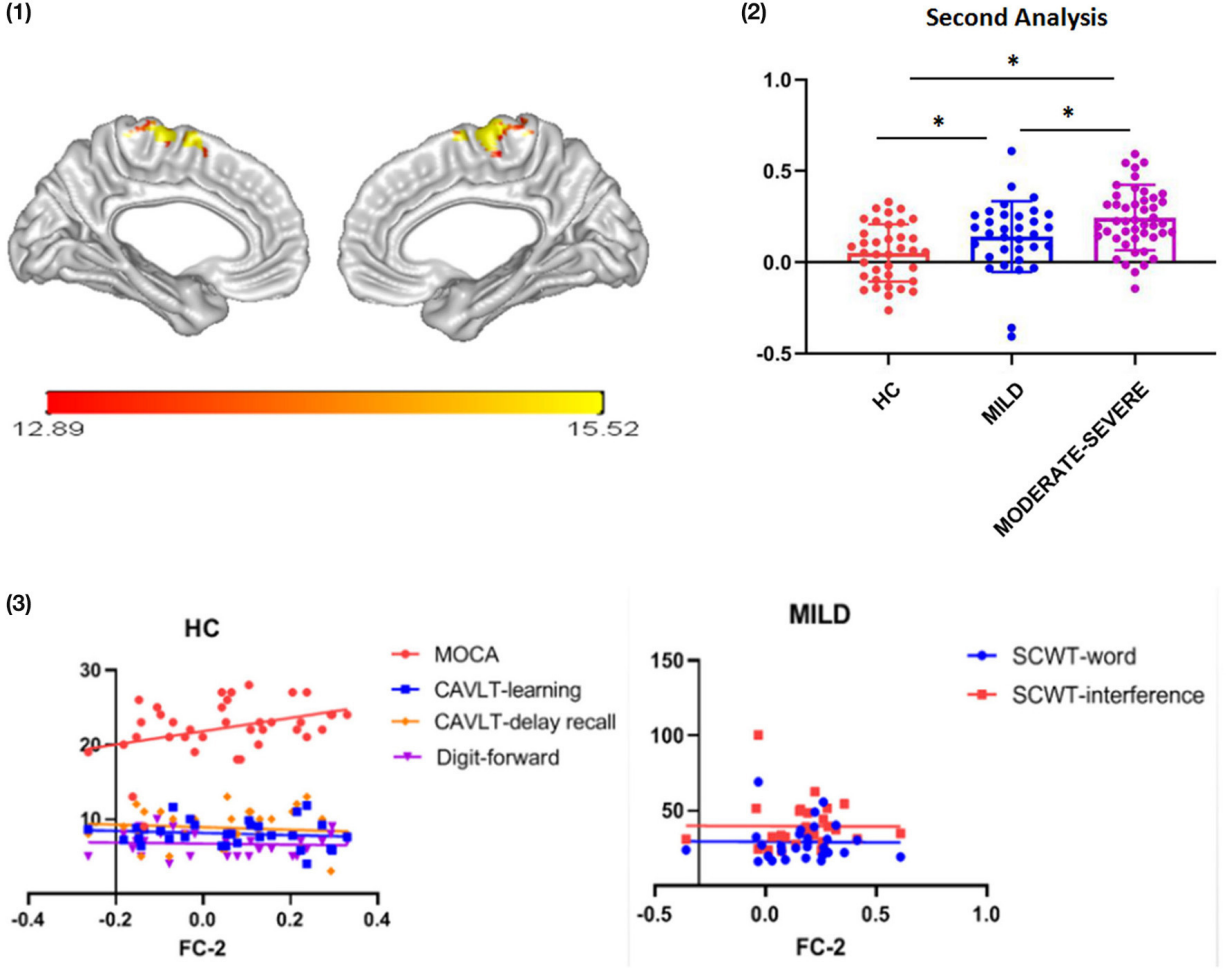
Functional connectivity between ITA and MFG. (1) ITA and MFG detected in thalamic subregion seed-whole brain analysis; (2) FC strength of ITA and MFG among the three groups; (3) Correlation between cognitive assessment scores and ITA-MFG FC strength. FC, functional connectivity; ITA, interthalamic adhesion; MOCA, Montreal cognitive assessment; CAVLT, Chinese auditory verbal learning test; SCWT, Stroop color and word test; WMHs, white matter hyperintensities; HC, healthy controls. *p < 0.05; error bars represent standard deviation.

In the second step, ITA was used as a seed to analyze the FC values between the thalamus and the whole cortex. Results showed that ITA-medial frontal gyrus (MFG) FC values were different among the three groups [Figure 3: (1)]. Compared with the HC group, the ITA-MFG FC values increased in the mild and moderate-severe WMH groups (*t* = 2.134, *p* = 0.036 and *t* = 5.197, *p* < 0.001, respectively). Importantly, ITA-MFG FC values in the moderate-severe WMH group increased compared with those in the mild WMH group [*t* = 2.480, *p* = 0.015; Figure 3: (2)]. Correlation analysis results suggested that ITA-MFG FC values were positively correlated with MOCA, CAVLT-learning, CAVLT-delay recall and DS-forward scores in the HC group (MOCA: *r* = 0.369, *p* = 0.025; CAVLT-learning: *r* = 0.375, *p* = 0.025; CAVLT-delay recall: *r* = 0.362, *p* = 0.042; DS-forward: *r* = 0.386, *p* = 0.027), whereas in the mild WMH group, ITA-MFG FC values were positively correlated with SCWT-word and SCWT-interference [*r* = 0.427, *p* = 0.021 and *r* = 0.421, *p* = 0.023, respectively; Figure 3: (3)].

## Discussion

In this study, we explored the abnormalities of the thalamocortical FC in WMH patients and its relationship with cognitive function. The main results were as follows: First, PrCG-ITA FC was increased greatly in the moderate-severe WMH group, and the values of FC were significantly related to cognitive function in each group. Second, ITA-MFG FC in the moderate-severe and mild WMH groups showed varying degrees of increase. At the same time, a positive correlation between ITA-MFG FC values and cognition was also found in mild and HC groups. Overall, these results highlighted the important role of thalamocortical FC in cognitive impairment of WMH patients. We also demonstrated that WMH patients had lower cognitive scores than the normal population in terms of overall cognitive function, as well as attention, execution, memory and language. Finally, the increase of thalamocortical FC in WMH patients may be a compensatory mechanism after white matter damage.

Cerebral small vessel disease is a collective term for many pathological types, which can be manifested as white matter hyperintensities, lacunes, microbleeds and so on. It is rarely to select patients only manifested as white matter hyperintensities, and may be combined with multiple kind of pathological changes. Therefore, we described lacunes and microbleeds in HC group, mild WMH group and moderate-severe WMH group (Table 2). The results showed that the number of lacunes in frontal lobe, basal ganglia and whole brain were more severe in the WMH group than in HC group. Besides, the lacunes of whole brain in moderate-severe group was significantly higher than that of the mild group. In terms of microbleeds, the number of moderate-severe group was significantly more than that of HC group, which also appeared in frontal lobe, basal ganglia, thalamus and whole brain (Table 2). In addition, demographic information results show that there are significantly more hypertensive patients in WMH group than in HC group. Hypertension is considered to be one of the most important factors by damaging the WMH (Pantoni et al., 1997; Xiong et al., 2011). Hypertension and white matter hyperintensities are closely linked to cognitive decline (Wolf, 2012; Peters et al., 2019) and associated with WMH progression (Verhaaren et al., 2013). Therefore, it is suggested that hypertensive patients in mild and moderate-severe WMH group are significantly higher than HC group. However, the numbers of hypertension in severe WMH group was not statistically different from that in mild group (Table 1). In addition, brain tissue is an early damage target organ for hypertension. It is probably that hyperintensities is one of the important mechanisms of cognitive impairment caused by hypertension. WMH volumes of WMH patients were also quantified by UBO detector in this study. It can be concluded that the periventricular white matter volumes of the moderate-severe WMH group were significantly greater than that of the mild WMH group. In addition, the total brain white matter volumes of the moderate-severe WMH group were significantly higher than that of the mild WMH group. But, there was no significant difference in deep white matter volumes between the two groups (Table 3).

**Table 2:** Description of lacunes and microbleeds in three groups.

	**Lacunes**			**Microbleeds**		
	**HC^**a**^**	**MILD^**b**^**	**Moderate-Severe^**c**^**	**HC^**a**^**	**MILD^**b**^**	**Moderate-Severe^**c**^**
	**(*n* = 32)**	**(*n* = 29)**	**(*n* = 47)**	**(*n* = 32)**	**(*n* = 25)**	**(*n* = 37)**
**Subcortical**					
Frontal	0 (0.0)	0 (0.1)	0 (0.2)	0 (0.1)	0 (0.1)	0 (0.3)
Parietal	0 (0.0)	0 (0.1)	0 (0.1)	0 (0.1)	0 (0.3)	0 (0.1)
Occipital	0 (0.0)	0 (0.0)	0 (0.1)	0 (0.1)	0 (0.1)	0 (0.4)
Temporal	0 (0.0)	0 (0.1)	0 (0.2)	0 (0.1)	0 (0.5)	0 (0.13)
Any subcortical	0 (0.0)	0 (0.0)	0 (0.1)	0 (0.0)	0 (0.1)	0 (0.4)
**Deep**					
Basal ganglia	0 (0.0)	0 (0.2)	0 (0.3)	0 (0.1)	0 (0.2)	0 (0.5)
Thalamus	0 (0.0)	0 (0.0)	0 (0.1)	0 (0.0)	0 (0.1)	0 (0.6)
Internal capsule	0 (0.0)	0 (0.0)	0 (0.1)	0 (0.0)	0 (0.3)	0 (0.7)
Any deep	0 (0.0)	0 (0.0)	0 (0.0)	0 (0.1)	0 (0.1)	0 (0.4)
**Infratentorial**					
Cerebellum	0 (0.0)	0 (0.0)	0 (0.1)	0 (0.1)	0 (0.1)	0 (0.16)
Pons	0 (0.0)	0 (0.1)	0 (0.0)	0 (0.0)	0 (0.2)	0 (0.2)
Mesencephalon	0 (0.0)	0 (0.0)	0 (0.0)	0 (0.1)	0 (0.2)	0 (0.3)
Medulla Oblongata	0 (0.0)	0 (0.0)	0 (0.0)	0 (0.0)	0 (0.1)	0 (0.5)
Total	0 (0.0)	0 (0.1)	0 (0.2)	0 (0.0.75)	0 (0.2)	1 (0.3)

a *Represents the HC group*.

b *Represents the Mild WMH group*.

c *Represents the Moderate-Severe WMH group*.

*Lacunes: Frontal: p (a-c) = 0.040; Basal ganglia: p (a-c) = 0.015; Total: p (b-c) = 0.039, p (a-c) < 0.001; Microbleeds: Frontal: p (a-c) = 0.037; Basal ganglia: p (a-c) = 0.009; Thalamus: p (a-c) = 0.023; Total: p (a-c) = 0.041; Non-normal distribution data is represented by median and interquartile range*.

**Table 3:** UBO detector-derived WMH volumes in mild and moderate-severe WMH group.

	**Mild WMH**	**Moderated-Severe WMH**	***t***	***p***
PVWMH volumes (mm^3^)	5,759.38 ± 3,073.10	13,901.84 ± 7,360.75	6.788	***<0.001***
DWMH volumes (mm^3^)	6,648.44 ± 12,457.04	9,397.07 ± 8,356.50	1.182	0.241
Total WMH volumes (mm^3^)	12,790.73 ± 13,276.32	24,749.59 ± 13,336.21	3.956	***<0.001***

*PVWMH, periventricular WMH; DWMH, deep WMH. p-values < 0.05 are labeled in bold and italic*.

Our findings suggested that thalamocortical circuits may be involved in the pathogenesis of WMH and are related to cognitive impairment. Several studies have implicated thalamocortical connections in wakefulness, arousal, attention and executive function. Damage to these connections has been recorded in children with cerebellar brain tumors and developmental disorders, and these connections are associated with deficits in working memory (Law et al., 2017) and decreased reading ability (Fan et al., 2014). Besides, one study mainly focused on fragile neural networks in pediatric focal epilepsy suggested that thalamocortical circuit damage is an important mechanism for impaired executive function in temporal lobe epilepsy (Law et al., 2018). In particular, another study found that in schizophrenia patients, the decrease in thalamic functional connectivity was combined with the prefrontal cortex, and the increase in thalamic functional connection was combined with the motor and somatosensory cortex (Keefe, 2014). It has also been reported that the functional connections of thalamus and sensorimotor areas were negatively related to higher cognitive functions, such as attention, vigilance, and processing speed in patients with schizophrenia (Chen P. et al., 2019). Taken together, these studies show that thalamocortical FC is inseparable from cognitive function and plays an important role in many diseases.

Recently, more and more evidence has shown that abnormalities in the thalamic cortical network may cause cognitive dysfunction in patients with WMH. One study on diffusion tensor imaging indicated that the brain structure network in WMH patients with cognitive impairment is seriously affected, and this disorder is relevant to the burden of WMH and cognitive deficits (Yang et al., 2020). It is reported that the amplitude of low-frequency fluctuations in the left parahippocampal gyrus was significantly reduced, and the amplitude of low-frequency fluctuations in the left inferior semi-lunar lobule and the right Supraorbital frontal gyrus increased of WMH patients. Besides, compared with HC group, WMH group showed an increased functional connectivity between the right insular region and right superior orbital frontal gyrus, and between the right calcarine cortex and left parahippocampal gyrus (Cheng et al., 2017). The results also indicate that abnormal thalamocortical FC is closely related with cognitive impairment in WMH patients.

ITA is an understudied neuroanatomical structure that has not been fully studied. It forms a bridge that connects the tissues of each hemisphere thalamus across the midline. However, its functional significance is still not totally understood. One group has shown that the gender difference in ITA size was related to the anatomical structure of the surrounding thalamus and neuropsychological functions, especially the attention function (Damle et al., 2017), which is also reflected in the current research. In addition, a research about fiber tractography had showed that the anterior thalamic radiation (ATR) consists of fibers between the frontal cortex and the mediodorsal thalamic nuclei (Zhou et al., 2003). The ATR is the mainly white matter bundle projection system within the brain that passes through the internal capsule, and is an important circuit component to form new memories. Impairment in executive functioning was most consistently linked to lesions in the dorsomedial nucleus (Sandson et al., 1991; Van der Werf et al., 2003), which is connected to the frontal cortex through the ATR (Biesbroek et al., 2013). In the current study, the close relationship between thalamocortical function and memory, as well as executive function, was also found.

Previous studies have shown that the PrCG, frontal gyrus and thalamus are all closely related to cognitive function. The PrCG is related to the inner sensory processing of motion and somatosensory information. Moreover, the atrophy of the PrCG may induced the decline of cognitive function in the elderly people, and led to the abnormality in mutual regulation function between the regulation of anticipatory threats and exercise avoidance support, and caused the abnormal network integration (Drabant et al., 2011; Ely et al., 2016; Di Bono et al., 2017). Through white matter fiber structure within brain, MFG is extensively connected with other cortical and subcortical areas. The PrCG is also connected to the ventral part of the MFG through the lower structure of the frontal longitudinal system, while the upper structure of the frontal longitudinal system connects the precentral gyrus with the dorsal part of the MFG (Catani et al., 2012). Importantly, the middle frontal lobe is traditionally closely related to other cognitive control processes, and its functions mainly include response selection, task switching and task set reconfiguration, prevention of interference and working memory (D'Esposito et al., 1995; Cohen et al., 2000; Dove et al., 2000; Brass and von Cramon, 2002; Chikazoe, 2010). Besides, one study hypothesized that the massive activation of brain regions in MFG was related to the combined results of Wisconsin card sorting task and task switching (Buchsbaum et al., 2005), which consistent with the results in our second FC analysis.

In summary, the above results indicated a robust thalamocortical signal transmission, in which ITA plays a role. The current study proved that the thalamocortical circuit was not only related to perception, but is also involved in the processing and transmission and integration of information, and is closely related to cognitive function. The results suggested that the abnormality of the thalamocortical circuit may be an important mechanism of cognitive impairment in patients with WMHs.

## Strengths and Limitations

We considered the relatively large number of participants in the study as a major strength; however, our study also had limitations. The effect of disease burden on FC in WMHs was only based on cross-sectional data. The lacunes and microbleeds also influence the performce of cognition in WMHs patients, which is another limitation of this study. In future research, we will start from the perspective of neuroimaging to further expand the sample size of diffusion tensor imaging data, and explore whether WMH patients also have an anatomical abnormality in thalamocortical functional connectivity, and clarify its relevance to cognitive impairment.

## Data Availability Statement

The raw data supporting the conclusions of this article will be made available by the authors, without undue reservation.

## Ethics Statement

This study involving human subjects were reviewed and approved by the Ethics Committee of the First Affiliated Hospital of Anhui Medical University. The patients/participants provided their written informed consent to participate in this study.

## Author Contributions

CC: collect and analyze data and write articles. XW, SC, ZW, and WP: collect data and statistical results. JZ: provide ideas and collect data. JY and BQ: measurement services. YT: provide ideas and recruit participants. QW and KW: guiding experimental ideas and assisting in writing articles. All authors contributed to the article and approved the submitted version.

## Conflict of Interest

The authors declare that the research was conducted in the absence of any commercial or financial relationships that could be construed as a potential conflict of interest.
